# Protein purification via consecutive histidine–polyphosphate interaction

**DOI:** 10.1002/pro.5021

**Published:** 2024-05-15

**Authors:** Zihao Zhou, Jin Jin, Xu Deng, Zongchao Jia

**Affiliations:** ^1^ School of Pharmaceutical Sciences Central South University Changsha Hunan China; ^2^ Department of Biomedical and Molecular Sciences Queen's University Kingston Ontario Canada

**Keywords:** histidine repeats, Ni‐NTA, polyphosphate, protein purification, resin

## Abstract

While nickel‐nitrilotriacetic acid (Ni‐NTA) has greatly advanced recombinant protein purification, its limitations, including nonspecific binding and partial purification for certain proteins, highlight the necessity for additional purification such as size exclusion and ion exchange chromatography. However, specialized equipment such as FPLC is typically needed but not often available in many laboratories. Here, we show a novel method utilizing polyphosphate (polyP) for purifying proteins with histidine repeats via non‐covalent interactions. Our study demonstrates that immobilized polyP efficiently binds to histidine‐tagged proteins across a pH range of 5.5–7.5, maintaining binding efficacy even in the presence of reducing agent DTT and chelating agent EDTA. We carried out experiments of purifying various proteins from cell lysates and fractions post‐Ni‐NTA. Our results demonstrate that polyP resin is capable of further purification post‐Ni‐NTA without the need for specialized equipment and without compromising protein activity. This cost‐effective and convenient method offers a viable approach as a complementary approach to Ni‐NTA.

## 1 INTRODUCTION

In the realm of recombinant protein purification, the emergence of nickel‐nitrilotriacetic acid (Ni‐NTA) has marked a significant breakthrough (Hochuli et al., 1987). Ni‐NTA, with its distinctive properties, is widely recognized for its versatility and efficiency in selectively binding to polyhistidine‐tagged proteins (Arnold, 1991; Hochuli et al., 1988). This specific interaction enables a highly targeted approach, streamlining the purification process and facilitating the isolation of the desired protein with high purity and yield. When Ni‐NTA fails to achieve satisfactory results in some cases, cobalt purification provides an alternative method with enhanced specificity and reduced risk of metal contamination, although more expensive (Zatloukalová & Kučerová, 2004).

Although Ni‐NTA has undoubtedly advanced protein purification, it grapples with several limitations. A notable challenge is the potential for nonspecific binding of contaminants that compromises the overall purity of the isolated protein (Gräslund et al., 2008; Spriestersbach et al., 2015). Additionally, the varying affinity of Ni‐NTA for polyhistidine tags among different proteins introduces unpredictability, impacting the selectivity of the purification process. For instance, immobilized carriers like sepharose beads possess a larger particle size, leading to an expanded surface area that reduces the effectiveness of the separation process. Therefore, additional purification techniques such as size exclusion and ionic exchange chromatography using FPLC become necessary, but their reliance on the specialized instrument often poses a challenge in many laboratories.

Ni‐NTA operates via an affinity interaction between Ni ions and histidine, necessitating a pH > 7, ideally within the range of pH 7–8. Since many proteins may have isoelectric points within this range (Kurotani et al., 2019; Tokmakov et al., 2021), the buffer must be outside of this pH range to prevent aggregation and precipitation, potentially compromising the efficacy of Ni‐NTA in purifying such proteins. Additionally, proteins often need to exist in a reduced state to maintain their structural and functional integrity, requiring the introduction of reducing agents, such as DTT. Unfortunately, DTT can lead to the reduction of Ni ions (Haumann et al., 2003), thereby diminishing the affinity of Ni‐NTA. In cases where EDTA is needed for specific protein applications, it interferes with Ni ions due to its chelating properties (Crowe et al., 1994). These combined constraints underscore the necessity for continuous efforts in exploring new and alternative purification methodologies.

Our lab recently made a serendipitous discovery related to polyphosphate (polyP, Figure S1) binding to proteins containing histidine‐repeat via non‐covalent interaction (Neville et al., 2023). PolyP, a linear polymer with inorganic phosphate residues, is found in all domains of life, ranging from bacterial to human cells (Kornberg et al., 1999), robustly binds to histidine‐repeat or histidine‐rich proteins (Malik et al., 2021), detectable through mobility shift on NuPAGE gel (Azevedo et al., 2015; Bentley‐DeSousa et al., 2018). PolyP‐histidine repeat interaction is highly specific; polyP does not bind to other biological polyanions, such as heparin, single‐stranded DNA, and dextran sulfate (Neville et al., 2023). PolyP functions as procoagulant through interacting with several proteins involved in blood clotting (Choi et al., 2011, 2015; Mutch et al., 2010), and its roles in other biological systems are also investigated by an expanding body of research (Han et al., 2007; Hernandez‐Ruiz et al., 2006; Kim & Cavanaugh, 2007; Morita et al., 2010; Pavlov et al., 2010). Our initial discovery originated from a protein containing a histidine‐tag during purification. Given that most recombinant proteins feature a polyhistidine purification tag, we hypothesized that His‐tag proteins could readily be purified through polyP interaction without additional purification tags or new constructs. Despite the relatively tight polyP binding, its non‐covalent nature allows salt‐dependent dissociation, enabling convenient decoupling and elution of target proteins from polyP interaction.

In this work, we demonstrate that immobilized polyP binds to proteins containing conventional His‐tags, which can be dissociated by a high‐salt solution. As a pilot study, we conducted polyP purification experiments on several proteins from both prokaryotic and eukaryotic sources. This novel method presents a promising advancement in protein purification without the need for additional constructs or alternative purification tags. It holds the potential to serve as a subsequent step after Ni‐NTA purification.

## 2 RESULTS AND DISCUSSION

### 2.1 Immobilization of polyP on amine‐activated agarose

In order to establish polyP as a viable purification method, our first objective was to immobilize polyP onto a suitable carrier. Building upon previous research (Choi et al., 2010), we opted to use amine‐activated agarose as the immobilization matrix for polyP (Figure S2). As depicted in Figure S3, after coupling with polyP overnight, in the toluidine blue assay the agarose resin exhibited a clear color change from blue to purple, indicating successful immobilization of polyP on amine‐activated agarose, thus enabling subsequent experiments.

### 2.2 Validation of His‐repeat proteins binding to polyP‐coupled resin

**FIGURE 1:**
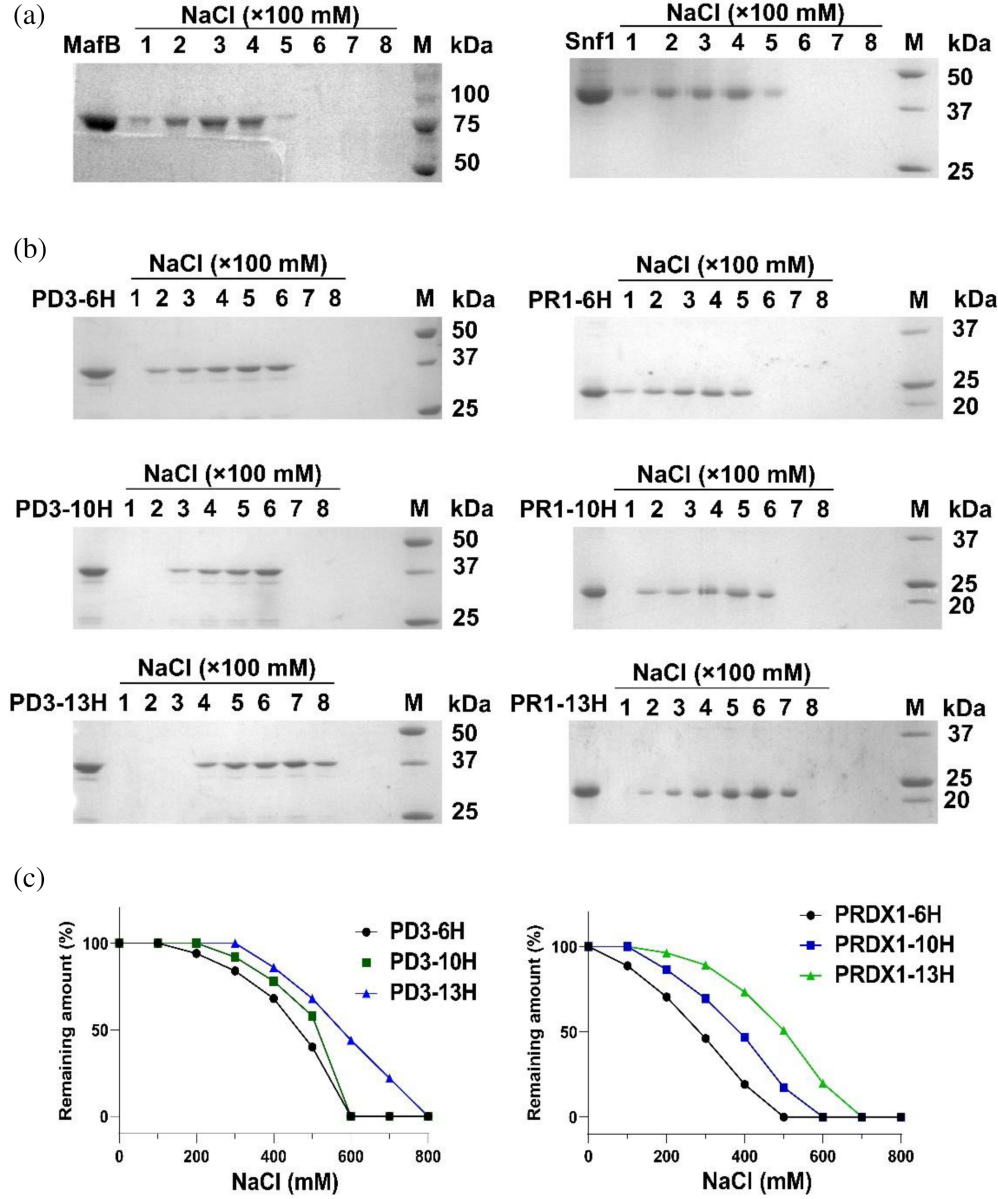
Validation of His‐repeat proteins binding with polyP resin. (a) PolyP resin binds to proteins containing internal His‐repeat in the wide‐type sequence. MafB: fully purified MBP‐MafB, Snf1: fully purified MBP‐Snf1. NaCl (×100 mM): wash solution containing the respective salt concentration (b) PolyP resin binds to proteins fused with 6×, 10×, and 13×His‐tags. PD3‐6H: fully purified PD3‐6H; PD3‐10H: fully purified PD3‐10H; PD3‐13H: fully purified PD3‐13H; PR1‐6H: fully purified PRDX1‐6H; PR1‐10H: fully purified PRDX1‐10H; PR1‐13H: fully purified PRDX1‐13H; NaCl (×100 mM): wash solution containing the respective salt concentration. (c) Binding efficiency of polyP resin to PD3 and PRDX1 in the presence of different concentrations of salt. Protein amount in SDS gel was quantified via ImageJ.

Although we have shown polyP binds to consecutive His‐containing proteins (Neville et al., 2023), we aimed to confirm whether this is also the case for the polyP‐coupled resin (referred to as polyP resin hereafter). We performed a binding assay of the polyP resin with human protein MafB (a transcription factor) and yeast protein Snf1 (AMP‐activated Ser/Thr protein kinase), both of which contain internal and consecutive histidines in their wild‐type sequences. Notably, proteins expressed from both constructs capable of binding to polyP in NuPAGE analysis do not have a 6xHis‐tag at any terminus (Neville et al., 2023). Prior to polyP binding experiment, they were fused with an MBP‐tag, then purified through amylose resin and size exclusion chromatography (SEC). Following an overnight incubation with polyP resin, we washed off both proteins from polyP resin using gradient concentration of salt. SDS–PAGE analysis indicated that both MBP‐MafB and MBP‐Snf1 were eluted gradually by increasing salt concentration, in agreement with the previous finding of non‐covalent binding of MafB and Snf1 to polyP (Neville et al., 2023) (Figure 1a).

Next, we sought to test whether purified His‐tagged proteins can bind to polyP resin using PD3 and PRDX1. PD3 is an alpha/beta hydrolase derived from *Brucella anthropi* (Gao, 2021), and PRDX1 is a peroxiredoxin‐1 protein from humans which catalyzes the reduction of hydrogen peroxide to water and alcohols (Chae et al., 2017). These proteins are engineered with 6x, 10x, or 13x His‐tags, respectively. Prior to polyP binding experiment, they were purified through Ni‐NTA and SEC. Our polyP binding results demonstrated all variants were capable of binding to polyP resin, with subsequent elution achieved via the salt gradient (Figure 1b). Notably, PD3‐13H required a higher concentration of NaCl (800 mM) for complete elution compared to PD3‐6H and PD3‐10H, which were completely eluted with 600 mM NaCl. PD3‐13H began to wash off the polyP resin with 400 mM salt, while PD3‐10H could be eluted with lower salt concentration (300 mM), and the elution of PD3‐6H commenced at 200 mM salt, indicating that PD3 with longer His‐tag exhibited a stronger binding to polyP resin. A similar trend was observed in the comparisons of PRDX1‐6H, PRDX1‐10H, and PRDX1‐13H, suggesting that the strength of the polyP‐protein binding positively correlates with the number of consecutive histidines in the protein sequence. Quantification of the amount of protein bound was performed by ImageJ (Figure 1c).

### 2.3 Assessment of the binding conditions

**FIGURE 2:**
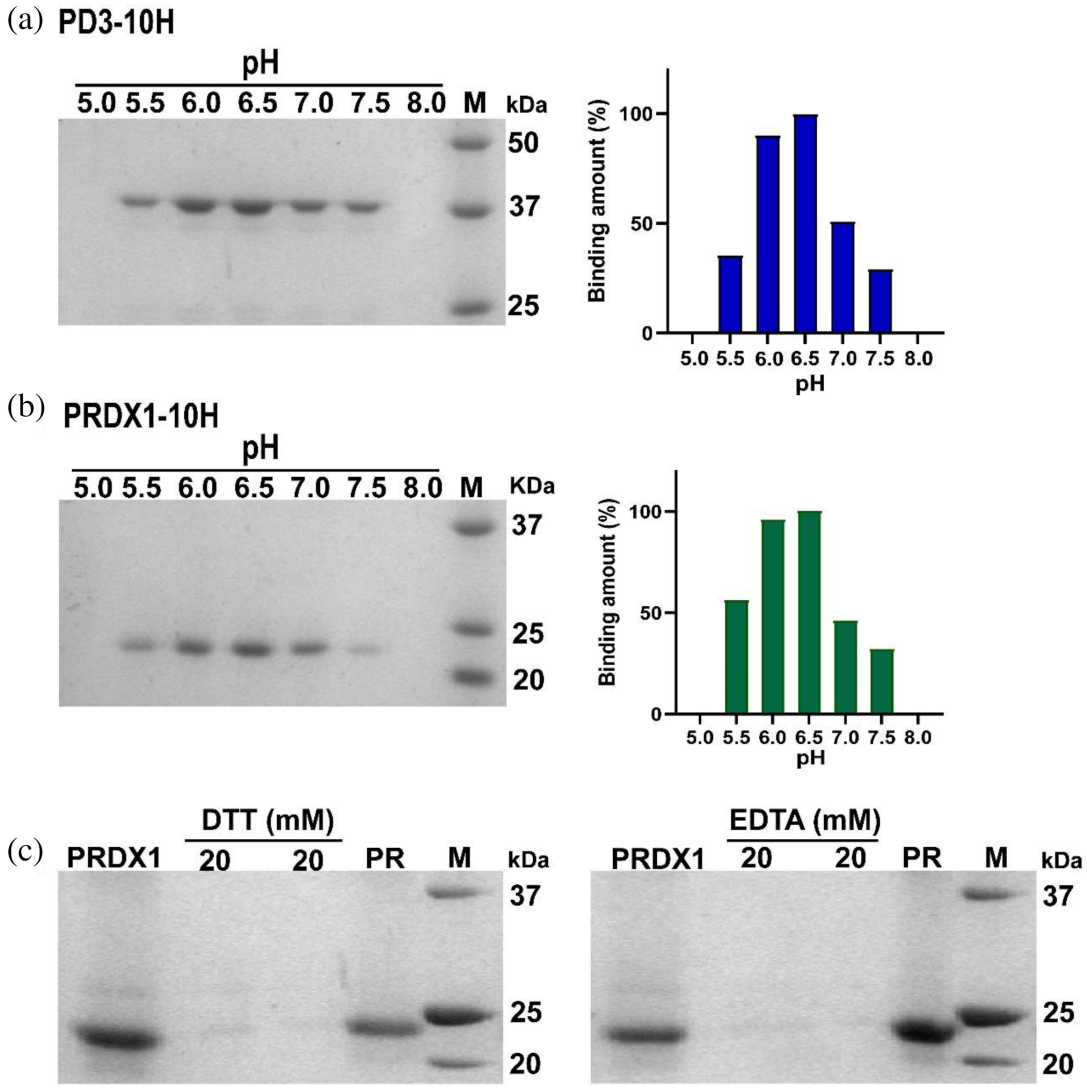
Evaluation of binding conditions of polyP resin. (a) The binding efficiency of polyP resin to PD3‐10H under different pH conditions. (b) The binding efficiency of polyP resin to PRDX1‐10H under different pH conditions. pH 5.0–8.0: polyP resin after overnight incubation with the proteins at the respective pH conditions. (c) Binding of polyP resin with PRDX1‐10H in the presence of DTT and EDTA. PRDX1, fully purified PRDX1‐10H binding to polyP resin; 20 mM DTT, wash solution containing 20 mM DTT; 20 mM EDTA, wash solution containing 20 mM EDTA; PR, polyP resin after overnight incubation with the PRDX1 and washed by 20 mM DTT or EDTA. The amount of protein was quantified by ImageJ.

Following the confirmation of polyP resin's binding capability to His‐tagged proteins, we screened the pH conditions for the resin's binding to PD3 and PRDX1, which were fully purified by Ni‐NTA and SEC. As depicted in Figure 2a,b, we observed that both PD3‐10H and PRDX1‐10H could bind to polyP resin within the pH range of 5.5–7.5, with the highest amount of bound protein observed at pH 6.5. Notably, no protein was detected on polyP resin at pH 5.0 and 8.0. This finding suggests that the polyP purification method can be applied within a pH range of 5.5–7.5.

Next, we assessed the tolerance of polyP resin to reducing and chelating agents. After incubating the resin and PRDX1‐10H and eluting with polyP binding buffer containing 20 mM DTT or EDTA, we observed that PRDX1 remained bound to the resin (Figure 2c). This indicates that DTT or EDTA up to 20 mM do not impact the protein binding to polyP resin. DTT help protein remain in the reduced state and avoid aggregation. EDTA is a common additive that mostly acts as a protease inhibitor and protects the protein from degradation.

### 2.4 Protein purification from cell lysate by polyP resin

**FIGURE 3:**
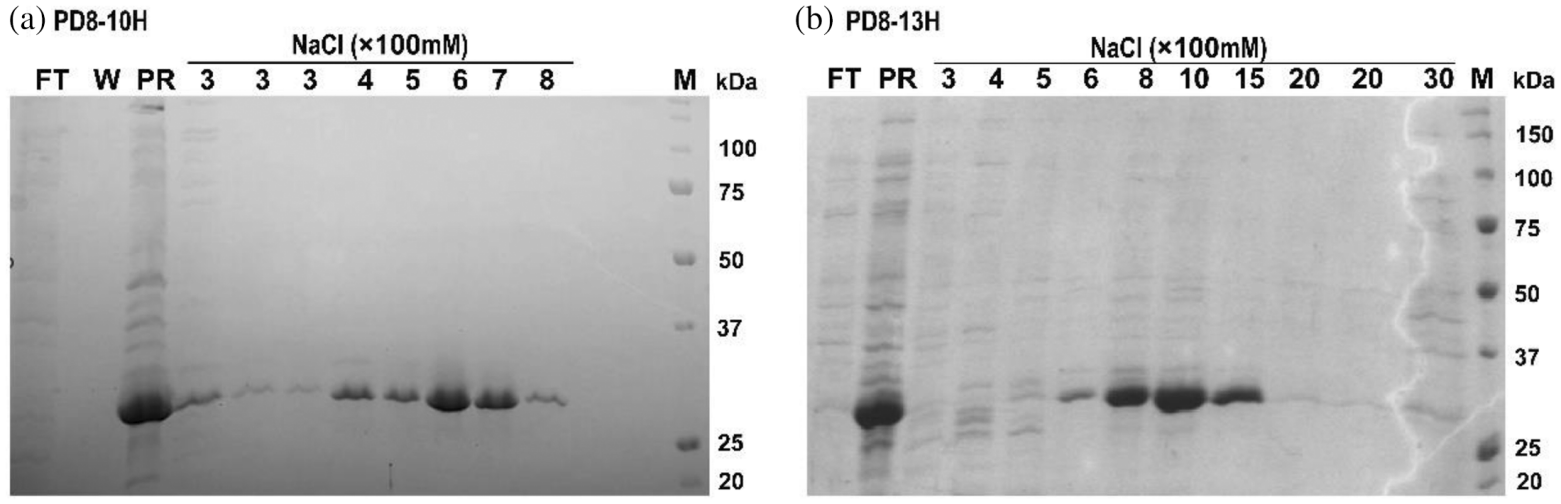
Protein purification from cell lysate using polyP resin. (a) Purification of PD8‐10H from the *E. coli* expression cell lysate. (b) Purification of PD8‐13H from the *E. coli* expression cell lysate. FT, flow through; PR, polyP resin after incubation with the lysate; W, wash solution without salt. NaCl (×100 mM), wash solution containing the respective salt concentration.

We first attempted to purify His‐tagged proteins directly from *E. coli* cell lysates using polyP resin. PD8, as well as PD5 in Section 2.5, are hydrolases from *Exiguobacterium* sp. (Gao, 2021). Expression cell lysate of PD8‐10H and PD8‐13H were incubated with polyP resin in the presence of 200 mM NaCl to reduce non‐specific binding and eluted using salt gradients. SDS–PAGE analysis indicated that the proteins could be largely purified, although minor impurities were observed (Figure 3). Unfortunately, after approximately 3–4 uses, polyP resin was gradually and reproducibly degraded, as evidenced by the gradual disappearance of violet color in the toluidine blue assay. Although the factor(s) in the cell lysates responsible for polyP breakdown is unknown, we suspect that small amounts of polyphosphatases from bacterial lysates like PPX (exopolyphosphatase) and Ppx/GppA (endopolyphosphatase) (Akiyama et al., 1993; Lonetti et al., 2011) may be the cause. If confirmed, this problem may be alleviated by engineering cell lines deficient in polyphosphatases.

Next, we incubated proteins with amine‐activated agarose resin only (i.e., without coupled polyP) and found that various proteins, including the target protein being purified, nonspecifically bound to the matrix in the absence of polyP. The varying amounts of bound protein, depending on the specific protein being purified, could lead to loss of protein yield. Unfortunately, the amine agarose resin is the only one commercially available. However, we believe that through screening different resin materials, a better support matrix can likely be found that minimizes nonspecific protein–matrix binding.

### 2.5 Purification of partially purified protein by polyP resin

**FIGURE 4:**
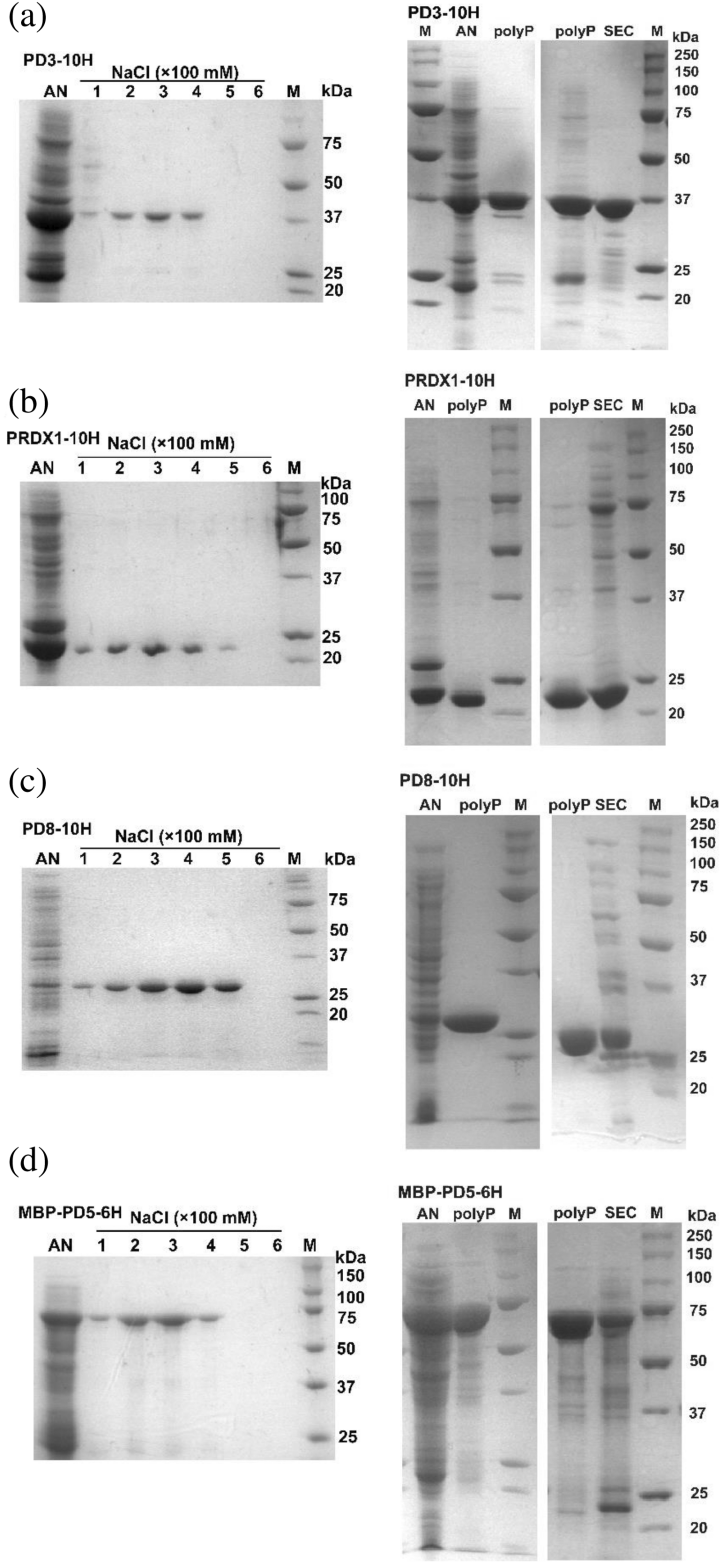
Post‐Ni‐NTA purification using polyP resin (left) and comparison between polyP and SEC purifications (right). (a) Purification of PD3‐10H. (b) Purification of PRDX1‐10H. (c) Purification of PD8‐10H. (d) Purification of MBP‐PD5‐6H. AN, pooled and concentrated impure fractions after Ni‐NTA purification; NaCl (×100 mM), wash solution containing the respective salt concentration; polyP, concentrated fractions purified via polyP resin; SEC, concentrated fractions purified from SEC. The same protein sample post Ni‐NTA purification was used for subsequent polyP and SEC purifications. Additionally, a similar total protein amount was employed in SDS–PAGE analysis to facilitate the comparison of purity and yield.

Due to polyP degradation when exposed to bacterial cell lysate, likely resulting from trace amounts of polyphosphatases, we next attempted to use Ni‐NTA to first remove or reduce the component(s) responsible for polyP degradation. In these experiments, we intentionally pooled partially purified protein fractions from Ni‐NTA elution and assessed the efficacy of polyP resin for protein purification. The impure protein fractions of PD3‐10H, PRDX1‐10H, PD8‐10H, and MBP‐PD5‐6H, were subjected to overnight incubation with polyP resin, followed by elution using salt concentration gradients. SDS–PAGE analysis indicated that protein purification was readily achieved via polyP resin for all proteins (Figure 4a–d). Notably, 10× and 13×His tagged proteins provided better purification compared to the 6xHis tag for most proteins.

Importantly, the impure samples resulting from the Ni‐NTA step did not degrade the polyP resin to the same extent as observed with cell lysates. PolyP was still detected in the resin via toludine blue assay after repeated use (Figure S4). This observation underscores the practical usefulness of the combined Ni‐NTA and polyP purification approach. Hence, in situations where proteins are not adequately purified by Ni‐NTA, particularly in cases where high purity is essential, such as for crystallization purposes, polyP resin purification can serve as a rebust and supplementary method for further protein purification post‐Ni‐NTA. We further compared the purity and yield of polyP purification and FPLC SEC purifications using partially purified proteins via Ni‐NTA. The best purity obtained was 96.8% from polyP and 82.5% from SEC, while the best recovery yield was 91.3% from polyP and 86.8% from SEC. The average purity obtained was ~86.8% from polyP and ~73.1% from SEC，while the average recovery yield was ~75.5% from polyP and ~84.2% from SEC (Table S2), which indicates polyP is comparable to SEC.

### 2.6 Esterase activity

To assess whether proteins purified via polyP resin retain their activity, we compared the esterase activity of the hydrolases, PD3‐10H, MBP‐PD5‐6H, and PD8‐10H, before and after polyP purification. As depicted in Figure S5, full activity was retained, indicating that polyP purification does not affect the proteins' integrity and activity.

## 3 MATERIALS AND METHODS

### 3.1 Immobilization of polyP onto amine‐activated agarose

The cross‐linking reagent, EDAC (1‐ethyl‐3‐[3‐(dimethylamino)propyl]carbodiimide), which can be used to couple primary amines to organic phosphates via stable phosphoramidate linkages (Hermanson, 2013), has been reported to effectively facilitate the covalent attachment of primary amine‐containing compounds and probe to the terminal phosphates of polyP via stable phosphoramidate bonds. Based on this property, we have coupled long‐chain polyP (polyP_700_) (Kerafast, USA) onto the amine‐activated agarose resin (Cube Biotech, Germany) via EDAC (Millipore Sigma, Germany). Briefly, 50 mg of amine‐activated agarose resin was incubated overnight at 4°C with 38 μM polyP_700_ (~700 phosphate units on average) in 100 mM MES, pH 6.2, 280 mM EDAC, and 1 mM CaCl_2_. Detection of the polyP resin was performed by adding a small amount of polyP resin into 100 μL of 20 μM toluidine blue (Millipore Sigma) (Sarabhai et al., 2015), resulting in a violet color that confirmed the successful coupling of polyP to the amine agarose.

### 3.2 Cloning and expression

The gene encoding MafB (Q9Y5Q3, *Homo sapiens*) were cloned into a custom pET16‐based vector called HT31 (MBP‐TEV‐protein), MBP‐tag fused Snf1 (P06782, *Saccharomyces cerevisiae*) and 6xhis‐MBP‐tag fused PD5 (QPI67555.1, *Exiguobacterium* sp.) were cloned into pET28a. 10×His‐tag fusion vector HT2‐10H and 13×His‐tag fusion vector HT2‐13H were constructed by inserting four more histidines and seven more histidines in hexahistidine gene of a custom pET21b‐based vector HT2 (protein‐6His). The genes encoding PD3 (QPA27412.1, *Brucella anthropi*), PD8 (QPI68425.1, *Exiguobacterium* sp.), and PRDX1 (Q06830, *Homo sapiens*) were cloned into the HT2, HT2‐10H, and HT2‐13H vectors, respectively.

Protein expression proceeded as follows: the cloned plasmids were transformed into *E. coli* BL21(DE3) RIPL competent cells (Agilent, Cat#230280) which were then spread onto culture plates containing appropriate antibiotics and incubated overnight at 37°C. Single colony of each transformant was picked from the plates and inoculated into 3 mL of LB medium supplemented with selective antibiotic, followed by overnight incubation at 37°C. Subsequently, 100 μL overnight LB culture was inoculated to 500 mL of Terrific Broth (TB) medium supplemented with selective antibiotic and incubated at 37°C in a shaker at 180 rpm until the optical density (OD_600_) reached 0.6. Expression was induced with 0.2 mM isopropyl β‐D‐1‐thiogalactopyranoside (IPTG) and incubated at 16°C for 12 h. The bacterial culture was harvested using a Beckman JS‐4.2 rotor at 4000 rpm for 30 min, and the resulting pellet was resuspended in 50 mL of lysis buffer (20 mM Tris–HCl, 150 mM NaCl, and 10% glycerol at pH 7.5) for post‐Ni‐NTA purification or a polyP binding buffer (5.6 mM Tris–HCl, 50 mM MOPS, pH 6.2, 5 mM Na_2_SO_4_) for direct purification of cell lysate. The resuspension was then frozen at −80°C for subsequent steps.

### 3.3 Protein purification via polyP resin

After resuspending cells, lysozyme and phenylmethylsulfonyl fluoride (PMSF) were added to the resuspended cells and incubated on ice for 30 min. Subsequently, cells were sonicated using a 5 s on and 15 s off pulse setting at 35% intensity for 6 min (Branson Sonifier, Model 450), soluble protein‐containing supernatants were obtained using a Beckman JA25.5 rotor at 18,000 rpm for 30 min. For protein purification via polyP resin directly from cell lysate, the cell lysate was prepared in the polyP binding buffer and the supernatant was incubated directly with 1 mL polyP resin at 4°C overnight, 200 mM NaCl was added during the incubation to minimize nonspecific binding. Elution was performed using gradient concentration of NaCl ranging from 300 to 1500 mM.

In the case of partially purified Ni‐NTA samples, the procedure is similar with the following exceptions. The culture pellet was resuspended in the lysis buffer. After sonication, the supernatant was incubated with 2 mL Ni‐NTA resin for 30 min at 4°C. The resin was subsequently washed with 250 mM imidazole wash buffer. Impure fractions containing target protein were collected and concentrated using a centrifugal filter (Millipore Sigma, Cat#UFC9050). The sample was exchanged to a polyP binding buffer via successive rounds of centrifugal filtration. After incubation with polyP resin, elution was carried out using gradient concentration of NaCl from 100 to 600 mM. SDS–PAGE (12% acrylamide/bis‐acrylamide) and protein bands were stained by Coomassie.

### 3.4 Esterase activity assay

The esterase activity of hydrolases, PD3‐10H, MBP‐PD5‐6H, and PD8‐10H before and after purification via polyP resin was determined on a 96‐well plate as previously (Nolasco‐Soria et al., 2018). The same quantity of hydrolases was used. The reaction mixture (100 mM K_3_PO_4_, pH 7.2, 1 μM hydrolase, and 0.5 mM substrate p‐nitrophenyl butyrate) was incubated at 37°C. Absorbance at 400 nm was monitored within 30 min (ε400 = 0.0148 M^−1^ cm^−1^). One unit of enzyme activity was defined as the release of 1.0 nanomole (10^−9^ mole) of p‐nitrophenol per minute at pH 7.2 and 37°C.

## 4 ADVANTAGES AND LIMITATIONS OF polyP PURIFICATION

Our work highlights several advantages of polyP purification: (1) as a complementary purification method to Ni‐NTA, polyP offers greater economy and convenience compared to FPLC; (2) polyP experiment is straightforward; (3) tolerant to commonly used chemicals in purification, such as DTT and EDTA; (4) no additional cloning is needed, although it is preferable to use a 10×His tag over the more common 6xHis tag. Key limitations include: (1) suboptimal purity, which may be addressed by exploring different resin materials; (2) polyP instability likely due to hydrolysis by polyphosphatases, leading to reduced protein binding; (3) slightly lower recovery yield compared to SEC; (4) elution using high concentrations of sodium chloride may cause precipitation for some proteins, although this rarely occurs.

## 5 CONCLUSION

In summary, our study reveals the utility of immobilized polyP for protein purification. Given the prevalence of His‐tagged recombinant proteins, this method offers a straightforward approach without requiring additional equipment or constructs. It is able to maintain protein activity after polyP purification. In the case of post‐Ni‐NTA purification, the polyP resin approach can serve as an alternative to size exclusion and ion exchange chromatography via FPLC. Furthermore, our proof‐of‐principle demonstration of direct protein purification from cell lysate sets the stage for future advancements. While not yet suitable for widespread application, our results in direct protein purification from cell lysate pave the way for future improvements. We anticipate the possibility of finding a superior solid matrix and preventing polyphosphate hydrolysis of polyP by engineering cell lines with selected polyphosphatase gene knockouts.

## AUTHOR CONTRIBUTIONS

**Zihao Zhou:** Writing – original draft; investigation; formal analysis; methodology; data curation; conceptualization; validation. **Jin Jin:** Data curation; conceptualization; investigation; writing – original draft; validation; methodology; formal analysis. **Xu Deng:** Supervision; project administration; writing – review and editing. **Zongchao Jia:** Writing – review and editing; writing – original draft; funding acquisition; resources; project administration; conceptualization; investigation; formal analysis; supervision; methodology.

## Supporting information


**APPENDIX S1:** Supporting information.

## References

[pro5021-bib-0001] Akiyama M , Crooke E , Kornberg A . An exopolyphosphatase of *Escherichia coli*. The enzyme and its ppx gene in a polyphosphate operon. J Biol Chem. 1993;268(1):633–639. 10.1016/S0021-9258(18)54198-3 8380170

[pro5021-bib-0002] Arnold FH . Metal‐affinity separations: a new dimension in protein processing. Bio/Technology. 1991;9(2):151–156. 10.1038/nbt0291-151 1369316

[pro5021-bib-0003] Azevedo C , Livermore T , Saiardi A . Protein polyphosphorylation of lysine residues by inorganic polyphosphate. Mol Cell. 2015;58(1):71–82. 10.1016/j.molcel.2015.02.010 25773596

[pro5021-bib-0004] Bentley‐DeSousa A , Holinier C , Moteshareie H , Tseng Y‐C , Kajjo S , Nwosu C , et al. A screen for candidate targets of lysine polyphosphorylation uncovers a conserved network implicated in ribosome biogenesis. Cell Rep. 2018;22(13):3427–3439. 10.1016/j.celrep.2018.02.104 29590613

[pro5021-bib-0005] Chae S , Lee H‐K , Kim Y‐K , Jung Sim H , Ji Y , Kim C , et al. Peroxiredoxin1, a novel regulator of pronephros development, influences retinoic acid and Wnt signaling by controlling ROS levels. Sci Rep. 2017;7(1):8874. 10.1038/s41598-017-09262-6 28827763 PMC5567039

[pro5021-bib-0006] Choi SH , Collins JNR , Smith SA , Davis‐Harrison RL , Rienstra CM , Morrissey JH . Phosphoramidate end labeling of inorganic polyphosphates: facile manipulation of polyphosphate for investigating and modulating its biological activities. Biochemistry. 2010;49(45):9935–9941. 10.1021/bi1014437 20957999 PMC2976836

[pro5021-bib-0007] Choi SH , Smith SA , Morrissey JH . Polyphosphate is a cofactor for the activation of factor XI by thrombin. Blood: J Am Soc Hematol. 2011;118(26):6963–6970. 10.1182/blood-2011-07-368811 PMC3245215 21976677

[pro5021-bib-0008] Choi SH , Smith SA , Morrissey JH . Polyphosphate accelerates factor V activation by factor XIa. Thromb Haemost. 2015;113(3):599–604. 10.1160/TH14-06-0515 25338662 PMC4541292

[pro5021-bib-0009] Crowe J , Dobeli H , Gentz R , Hochuli E , Stiiber D , Henco K . 6xffis‐Ni‐NTA chromatography as a superior technique in recombinant protein expression/purification. Protocols Gene Anal. 1994;31:371–387. 10.1385/0-89603-258-2:371 7921034

[pro5021-bib-0010] Gao R . A marine bacterial community capable of degrading poly (ethylene terephthalate) and polyethylene. J Hazard Mater. 2021;416:125928. 10.1016/j.jhazmat.2021.125928 34489083

[pro5021-bib-0011] Gräslund S , Nordlund P , Weigelt J , Hallberg BM , Bray J , Gileadi O , et al. Protein production and purification. Nat Methods. 2008;5(2):135–146. 10.1038/nmeth.f.202 pmc3178102. 18235434 PMC3178102

[pro5021-bib-0012] Han KY , Hong BS , Yoon YJ , Yoon CM , Kim Y‐K , Kwon Y‐G , et al. Polyphosphate blocks tumour metastasis via anti‐angiogenic activity. Biochem J. 2007;406(1):49–55. 10.1042/BJ20061542 17492939 PMC1948993

[pro5021-bib-0013] Haumann M , Porthun A , Buhrke T , Liebisch P , Meyer‐Klaucke W , Friedrich B , et al. Hydrogen‐induced structural changes at the nickel site of the regulatory [NiFe] hydrogenase from *Ralstonia eutropha* detected by X‐ray absorption spectroscopy. Biochemistry. 2003;42(37):11004–11015. 10.1021/bi034804d 12974636

[pro5021-bib-0014] Hermanson GT . Bioconjugate techniques. USA: Academic Press; 2013.

[pro5021-bib-0015] Hernandez‐Ruiz L , Gonzalez‐Garcia I , Castro C , Brieva JA , Ruiz FA . Inorganic polyphosphate and specific induction of apoptosis in human plasma cells. Haematologica. 2006;91(9):1180–1186. 16956816

[pro5021-bib-0016] Hochuli E , Döbeli H , Schacher A . New metal chelate adsorbent selective for proteins and peptides containing neighbouring histidine residues. J Chromatogr A. 1987;411:177–184. 10.1016/S0021-9673(00)93969-4 3443622

[pro5021-bib-0017] Hochuli E , Bannwarth W , Döbeli H , Gentz R , Stüber D . Genetic approach to facilitate purification of recombinant proteins with a novel metal chelate adsorbent. Bio/Technology. 1988;6(11):1321–1325. 10.1038/nbt1188-1321

[pro5021-bib-0018] Kim D , Cavanaugh EJ . Requirement of a soluble intracellular factor for activation of transient receptor potential A1 by pungent chemicals: role of inorganic polyphosphates. J Neurosci. 2007;27(24):6500–6509. 10.1523/JNEUROSCI.0623-07.2007 17567811 PMC6672444

[pro5021-bib-0019] Kornberg A , Rao NN , Ault‐Riché D . Inorganic polyphosphate: a molecule of many functions. Annu Rev Biochem. 1999;68(1):89–125. 10.1146/annurev.biochem.68.1.89 10872445

[pro5021-bib-0020] Kurotani A , Tokmakov AA , Sato K‐I , Stefanov VE , Yamada Y , Sakurai T . Localization‐specific distributions of protein pI in human proteome are governed by local pH and membrane charge. BMC Mol Cell Biol. 2019;20:1–10. 10.1186/s12860-019-0221-4 31429701 PMC6701068

[pro5021-bib-0021] Lonetti A , Szijgyarto Z , Bosch D , Loss O , Azevedo C , Saiardi A . Identification of an evolutionarily conserved family of inorganic polyphosphate endopolyphosphatases. J Biol Chem. 2011;286(37):31966–31974. 10.1074/jbc.M111.266320 21775424 PMC3173201

[pro5021-bib-0022] Malik RA , Zhou J , Fredenburgh JC , Truong TK , Crosby JR , Revenko AS , et al. Polyphosphate‐induced thrombosis in mice is factor XII dependent and is attenuated by histidine‐rich glycoprotein. Blood Adv. 2021;5(18):3540–3551. 10.1182/bloodadvances.2021004567 34474475 PMC8945577

[pro5021-bib-0023] Morita K , Doi K , Kubo T , Takeshita R , Kato S , Shiba T , et al. Enhanced initial bone regeneration with inorganic polyphosphate‐adsorbed hydroxyapatite. Acta Biomater. 2010;6(7):2808–2815. 10.1016/j.actbio.2009.12.055 20056175

[pro5021-bib-0024] Mutch NJ , Myles T , Leung LLK , Morrissey JH . Polyphosphate binds with high affinity to exosite II of thrombin. J Thromb Haemost. 2010;8(3):548–555. 10.1111/j.1538-7836.2009.03723.x 20002544 PMC2856763

[pro5021-bib-0025] Neville N , Lehotsky K , Yang Z , Klupt KA , Denoncourt A , Downey M , et al. Modification of histidine repeat proteins by inorganic polyphosphate. Cell Rep. 2023;42(9):113082. 10.1016/j.celrep.2023.113082 37660293

[pro5021-bib-0026] Nolasco‐Soria H , Moyano‐López F , Vega‐Villasante F , del Monte‐Martínez A , Espinosa‐Chaurand D , Gisbert E , et al. Lipase and phospholipase activity methods for marine organisms. Lipases and phospholipases: methods and protocols: 1835. USA: Humana Press; 2018. p. 139–167. 10.1007/978-1-4939-8672-9_7 30109650

[pro5021-bib-0027] Pavlov E , Aschar‐Sobbi R , Campanella M , Turner RJ , Gómez‐García MR , Abramov AY . Inorganic polyphosphate and energy metabolism in mammalian cells. J Biol Chem. 2010;285(13):9420–9428. 10.1074/jbc.M109.013011 20124409 PMC2843191

[pro5021-bib-0028] Sarabhai S , Harjai K , Sharma P , Capalash N . Ellagic acid derivatives from *Terminalia chebula* Retz. increase the susceptibility of *Pseudomonas aeruginosa* to stress by inhibiting polyphosphate kinase. J Appl Microbiol. 2015;118(4):817–825. 10.1111/jam.12733 25640983

[pro5021-bib-0029] Spriestersbach A , Kubicek J , Schäfer F , Block H , Maertens B . Chapter one—Purification of his‐tagged proteins. In: Lorsch JR , editor. Methods in enzymology. Volume 559. USA: Academic Press; 2015. p. 1–15. 26096499 10.1016/bs.mie.2014.11.003

[pro5021-bib-0030] Tokmakov AA , Kurotani A , Sato K‐I . Protein pI and intracellular localization. Front Mol Biosci. 2021;8:775736. 10.3389/fmolb.2021.775736 34912847 PMC8667598

[pro5021-bib-0031] Zatloukalová E , Kučerová Z . Separation of cobalt binding proteins by immobilized metal affinity chromatography. J Chromatogr B. 2004;808(1):99–103. 10.1016/j.jchromb.2004.03.066 15236692

