# Supplementary material for: Protein purification via consecutive histidine–polyphosphate interaction
Source: Protein Sci. 2024 May 15;33(6):e5021. doi: 10.1002/pro.5021 (PMC11094774; doi:10.1002/pro.5021)
Supplement: Supplementary file 1 — APPENDIX S1: Supporting information. [file PRO-33-e5021-s001.docx]

**Supplementary Information**

**Protein Purification via Consecutive Histidine-Polyphosphate Interaction**

Zihao Zhou^1,2,3^, Jin Jin^2,3^, Xu Deng^1^ and Zongchao Jia^2,4^

^1^School of Pharmaceutical Sciences, Central South University, Changsha, Hunan, China

^2^Department of Biomedical and Molecular Sciences, Queen’s University, Kingston, Ontario, Canada

^3^Equal contribution

^4^Corresponding author

**Contact information of corresponding author**

Department of Biomedical and Molecular Sciences,

Queen’s University,

18 Stuart Street, Kingston,

ON KL7 3N6, Canada

Tel: 613 533-6277

E-mail: [jia@queensu.ca](mailto:jia@queensu.ca)

##

##

**Figure S1.** The chemical structure of polyphosphate (n = 3 - ~1000).

**Figure S2.** The coupling reaction and immobilization of polyP on amine-activated resin.


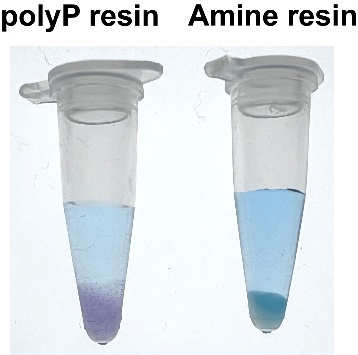


## Figure S3. The polyP resin turned into violet in the toluidine blue assay after successful polyP coupling to the amine-activated agarose matrix.


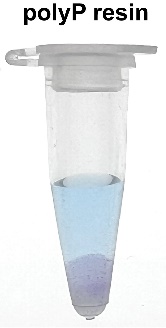


## Figure S4. After repeated post-Ni-NTA purifications, a persistent violet hue was observed in the toluidine blue assay, indicative of retained polyP within the matrix. However, it is notable that the intensity of the violet color appeared diminished compared to the initial resin (as shown in Figure S3, left vial), implying a gradual loss of polyP.

##
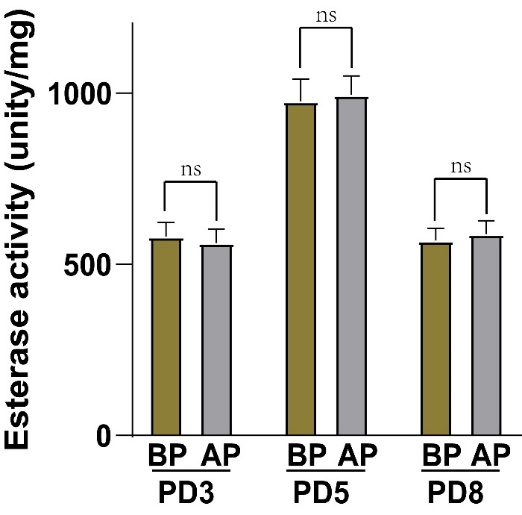


Figure S5. The esterase activity of PD3-10H, MBP-PD5-6H, and PD8-10H before and after purification by polyP resin. The same quantity of protein was used. BP: activity before purification via polyP resin; AP: activity after purification via polyP resin. Images are representative of n=3 technical replicates.


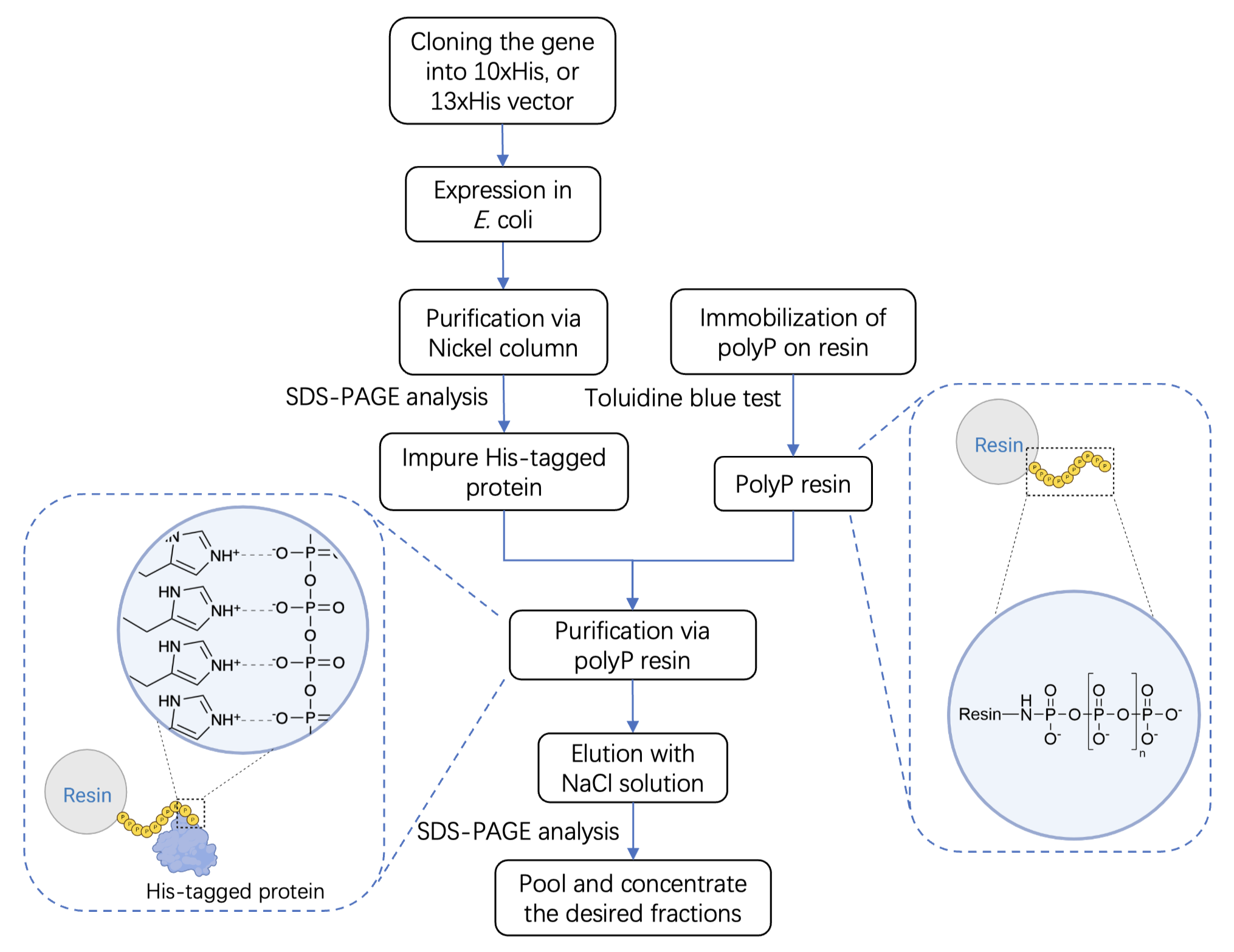


**Figure S6**. The schematic of polyP purification.

## Table S1. Summary of proteins used in this work.

| Protein | Source | Identifier | Function |
| --- | --- | --- | --- |
| MafB | Human | Q9Y5Q3 | Transcription factor |
| Snf1 | Yeast: *Saccharomyces cerevisiae* | P06782 | AMP-activated S/T protein kinase |
| PD3 | Bacterium: *Brucella anthropi* | QPA27412.1 | alpha/beta hydrolase |
| PD5 | Bacterium: *Exiguobacterium* sp. | QPI67555.1 | G-D-S-L family lipolytic protein |
| PD8 | Bacterium: *Exiguobacterium* sp. | QPI68425.1 | alpha/beta hydrolase |
| PRDX1 | Human | Q06830 | peroxiredoxin-1 |

## Table S2. Quantification and comparison of polyP and SEC purification using partially purified proteins. The amount of the proteins was measured using Bradford assay. The purity was quantified using Image Lab 6.2 software. Input: the sample loaded on polyP beads or SEC column; Output: the sample purified by polyP purification or SEC.

|  |  | **Input** | | **Output** | | **Recovery of tagged protein** |
| --- | --- | --- | --- | --- | --- | --- |
|  |  | **Total protein** | **Purity** | **Total protein** | **Purity** |  |
| **PD3-10H** | **NiNTA-polyP** | 8mg | 43.2% | 2.77mg | 75.8% | 60.8% |
|  | **NiNTA-SEC** | 8mg | 43.2% | 3.5mg | 82.5% | 83.5% |
| **MBP-PD5-6H** | **NiNTA-polyP** | 8.9mg | 38.7% | 3.21mg | 79.5% | 73.9% |
|  | **NiNTA-SEC** | 8.9mg | 38.7% | 4.45mg | 62.7% | 81.0% |
| **PD8-10H** | **NiNTA-polyP** | 12mg | 29.8% | 3.37mg | 96.8% | 91.3% |
|  | **NiNTA-SEC** | 12mg | 29.8% | 4.23mg | 72.3% | 85.5% |
| **PRDX1-10H** | **NiNTA-polyP** | 7mg | 53.6% | 3.01mg | 95.2% | 76.1% |
|  | **NiNTA-SEC** | 7mg | 53.6% | 4.36mg | 74.7% | 86.8% |

## Table S3. Sequence of various protein constructs.

| Protein | Sequence | Molecular weight (kDa) |
| --- | --- | --- |
| MBP-MafB | MKIEEGKLVIWINGDKGYNGLAEVGKKFEKDTGIKVTVEHPDKLEEKFPQVAATGDGPDIIFWAHDRFGGYAQSGLLAEITPDKAFQDKLYPFTWDAVRYNGKLIAYPIAVEALSLIYNKDLLPNPPKTWEEIPALDKELKAKGKSALMFNLQEPYFTWPLIAADGGYAFKYENGKYDIKDVGVDNAGAKAGLTFLVDLIKNKHMNADTDYSIAEAAFNKGETAMTINGPWAWSNIDTSKVNYGVTVLPTFKGQPSKPFVGVLSAGINAASPNKELAKEFLENYLLTDEGLEAVNKDKPLGAVALKSYEEELAKDPRIAATMENAQKGEIMPNIPQMSAFWYAVRTAVINAASGRQTVDEALKDAQTNSPSGGENLYFQGFGSAAELSMGPELPTSPLAMEYVNDFDLLKFDVKKEPLGRAERPGRPCTRLQPAGSVSSTPLSTPCSSVPSSPSFSPTEQKTHLEDLYWMASNYQQMNPEALNLTPEDAVEALIGSHPVPQPLQSFDSFRGAHHHHHHHHPHPHHAYPGAGVAHDELGPHAHPHHHHHHQASPPPSSAASPAQQLPTSHPGPGPHATASATAAGGNGSVEDRFSDDQLVSMSVRELNRHLRGFTKDEVIRLKQKRRTLKNRGYAQSCRYKRVQQKHHLENEKTQLIQQVEQLKQEVSRLARERDAYKVKCEKLANSGFREAGSTSDSPSSPEFFL | 76.4 |
| MBP-Snf1 (1-65) | MKIEEGKLVIWINGDKGYNGLAEVGKKFEKDTGIKVTVEHPDKLEEKFPQVAATGDGPDIIFWAHDRFGGYAQSGLLAEITPDKAFQDKLYPFTWDAVRYNGKLIAYPIAVEALSLIYNKDLLPNPPKTWEEIPALDKELKAKGKSALMFNLQEPYFTWPLIAADGGYAFKYENGKYDIKDVGVDNAGAKAGLTFLVDLIKNKHMNADTDYSIAEAAFNKGETAMTINGPWAWSNIDTSKVNYGVTVLPTFKGQPSKPFVGVLSAGINAASPNKELAKEFLENYLLTDEGLEAVNKDKPLGAVALKSYEEELAKDPRIAATMENAQKGEIMPNIPQMSAFWYAVRTAVINAASGRQTVDAALAAAQTNAAAGSMSSNNNTNTAPANANSSHHHHHHHHHHHHHGHGGSNSTLNNPKSSLADGAHIGNYQIVKTLGEGS | 47.6 |
| PD3-6H | MGSMFRFHIVSALLTLFIAVPSQAHDVGQREIKISGAEPGRNLEVSVWYPAAIDGKATLIGDNIIFKGAPAVIDATPEKGSFPLLVMSHGSGGRAQGMAWLATELVKAGIIVAAPNHPGTTSGDSTPQDTPKIWQRTGDLSAVIDTMTTDPAWSGIIDKNKISVLGFSLGGAAAMEIAGARANLEAYARYCDTYGKWDCAWYAGGIGYRDDKQIKVDKVDLRSVDKSRFEQSNLDRRIASAIMIDPGLAQAYDAQSLASISIPMSFINLGEVETIPTGVVADKLASITPQGTYVTVAGATHFSFLPECKKGAADRLKSAGEVDPICSDDARRPRADIHADITKLVLKALQSMPKTLEHHHHHH | 38.8 |
| PD3-10H | MGSMFRFHIVSALLTLFIAVPSQAHDVGQREIKISGAEPGRNLEVSVWYPAAIDGKATLIGDNIIFKGAPAVIDATPEKGSFPLLVMSHGSGGRAQGMAWLATELVKAGIIVAAPNHPGTTSGDSTPQDTPKIWQRTGDLSAVIDTMTTDPAWSGIIDKNKISVLGFSLGGAAAMEIAGARANLEAYARYCDTYGKWDCAWYAGGIGYRDDKQIKVDKVDLRSVDKSRFEQSNLDRRIASAIMIDPGLAQAYDAQSLASISIPMSFINLGEVETIPTGVVADKLASITPQGTYVTVAGATHFSFLPECKKGAADRLKSAGEVDPICSDDARRPRADIHADITKLVLKALQSMPKTEFLEHHHHHHHHHH | 39.4 |
| PD3-13H | MGSMFRFHIVSALLTLFIAVPSQAHDVGQREIKISGAEPGRNLEVSVWYPAAIDGKATLIGDNIIFKGAPAVIDATPEKGSFPLLVMSHGSGGRAQGMAWLATELVKAGIIVAAPNHPGTTSGDSTPQDTPKIWQRTGDLSAVIDTMTTDPAWSGIIDKNKISVLGFSLGGAAAMEIAGARANLEAYARYCDTYGKWDCAWYAGGIGYRDDKQIKVDKVDLRSVDKSRFEQSNLDRRIASAIMIDPGLAQAYDAQSLASISIPMSFINLGEVETIPTGVVADKLASITPQGTYVTVAGATHFSFLPECKKGAADRLKSAGEVDPICSDDARRPRADIHADITKLVLKALQSMPKTEFLEHHHHHHHHHHHHH | 39.8 |
| MBP-PD5-6H | MHHHHHHMKIEEGKLVIWINGDKGYNGLAEVGKKFEKDTGIKVTVEHPDKLEEKFPQVAATGDGPDIIFWAHDRFGGYAQSGLLAEITPDKAFQDKLYPFTWDAVRYNGKLIAYPIAVEALSLIYNKDLLPNPPKTWEEIPALDKELKAKGKSALMFNLQEPYFTWPLIAADGGYAFKYENGKYDIKDVGVDNAGAKAGLTFLVDLIKNKHMNADTDYSIAEAAFNKGETAMTINGPWAWSNIDTSKVNYGVTVLPTFKGQPSKPFVGVLSAGINAASPNKELAKEFLENYLLTDEGLEAVNKDKPLGAVALKSYEEELAKDPRIAATMENAQKGEIMPNIPQMSAFWYAVRTAVINAASGRQTVDAALAAAQTNAAAENLYFQGGSMKQSKWYAFLSGAVLLTGIAVYGLWIGYQDIVNPPERTLSVSEDERPEPEGDVYVALGDSLTRGVGSTSGAGYVQPVSTALEEEGVRTQNLAVSGARTEDLLTQLEQPEVRRTIENARYITLTIGGNDLFNRGENVDNFESVDIQQVVTDAKTNLETIFTEIRSLNDSATIVYIGLYNPFQNDENGQAFNQLILDWNASAKQLANAQNIDVIDPFAYISDLSRDLATDQFHPSDRTYEKFANDVLFVLQ | 70.1 |
| PD8-10H | MGSMMGYITAHDGTNLYVEDVGFGEPIVFLHGWPANNNMFEYQKNALVEAGYRYIGIDFRGYGKSDAPATGYDYETMASDVHMVVYGLGLKNFTLVGFSMGGAIAVRYAADYKEDGLNKLVLAGAAAPIFTQRADYPYGMTTDEVDALIDDTRADRPKMLEGFGEIFFEKEHSKPMQNWFHHLALVASSHGTIASAQALRDEDLRDALPKIDVETLIIHGVHDKICPFAFAEQMEQGISNARIERFEESGHGTVLDEREKFNDTLLQFVQLEHHHHHHHHHH | 31.7 |
| PD8-13H | MGSMMGYITAHDGTNLYVEDVGFGEPIVFLHGWPANNNMFEYQKNALVEAGYRYIGIDFRGYGKSDAPATGYDYETMASDVHMVVYGLGLKNFTLVGFSMGGAIAVRYAADYKEDGLNKLVLAGAAAPIFTQRADYPYGMTTDEVDALIDDTRADRPKMLEGFGEIFFEKEHSKPMQNWFHHLALVASSHGTIASAQALRDEDLRDALPKIDVETLIIHGVHDKICPFAFAEQMEQGISNARIERFEESGHGTVLDEREKFNDTLLQFVQLEHHHHHHHHHHHHH | 32.1 |
| PRDX1-6H(1-175) | MGRGSHHHHHHGGENLYFQGGPGSPNSMSSGNAKIGHPAPNFKATAVMPDGQFKDISLSDYKGKYVVFFFYPLDFTFVSPTEIIAFSDRAEEFKKLNCQVIGASVDSHFSHLAWVNTPKKQGGLGPMNIPLVSDPKRTIAQDYGVLKADEGISFRGLFIIDDKGILRQITVNDLPVGRSVDETLRLVQAFQFTDKHGEVCPA | 22.2 |
| PRDX1-10H(1-175) | MGRGSHHHHHHHHHHGGENLYFQGGPGSPNSMSSGNAKIGHPAPNFKATAVMPDGQFKDISLSDYKGKYVVFFFYPLDFTFVSPTEIIAFSDRAEEFKKLNCQVIGASVDSHFSHLAWVNTPKKQGGLGPMNIPLVSDPKRTIAQDYGVLKADEGISFRGLFIIDDKGILRQITVNDLPVGRSVDETLRLVQAFQFTDKHGEVCPA | 22.7 |
| PRDX1-13H(1-175) | MGRGSHHHHHHHHHHHHHGGENLYFQGGPGSPNSMSSGNAKIGHPAPNFKATAVMPDGQFKDISLSDYKGKYVVFFFYPLDFTFVSPTEIIAFSDRAEEFKKLNCQVIGASVDSHFSHLAWVNTPKKQGGLGPMNIPLVSDPKRTIAQDYGVLKADEGISFRGLFIIDDKGILRQITVNDLPVGRSVDETLRLVQAFQFTDKHGEVCPA | 23.1 |

*Maltose binding protein (MBP), linker region, TEV cleavage site, 6x, 10x, or 13xHis
